# Closed-Loop Transcutaneous Auricular Vagal Nerve Stimulation: Current Situation and Future Possibilities

**DOI:** 10.3389/fnhum.2021.785620

**Published:** 2022-01-04

**Authors:** Yutian Yu, Jing Ling, Lingling Yu, Pengfei Liu, Min Jiang

**Affiliations:** ^1^Acupuncture Department, Beijing Shijitan Hospital, Capital Medical University, Beijing, China; ^2^Ninth School of Clinical Medicine, Peking University, Beijing, China; ^3^Department of Gynecology, Shenzhen Traditional Chinese Medicine Hospital, Shenzhen, China; ^4^Department of Chinese and Western Medicine, Tongji Hospital, Tongji Medical College, Huazhong University of Science and Technology, Wuhan, China; ^5^Department of Anesthesiology, Beijing Shijitan Hospital, Capital Medical University, Beijing, China

**Keywords:** closed-loop (CL), transcutaneous auricular vagal nerve stimulation (taVNS), electromyography (EMG), electroencephalography (EEG), electrocardiography (ECG), subcutaneous humoral signals (SHS), non-invasive brain stimulation (NIBS)

## Abstract

Closed-loop (CL) transcutaneous auricular vagal nerve stimulation (taVNS) was officially proposed in 2020. This work firstly reviewed two existing CL-taVNS forms: motor-activated auricular vagus nerve stimulation (MAAVNS) and respiratory-gated auricular vagal afferent nerve stimulation (RAVANS), and then proposed three future CL-taVNS systems: electroencephalography (EEG)-gated CL-taVNS, electrocardiography (ECG)-gated CL-taVNS, and subcutaneous humoral signals (SHS)-gated CL-taVNS. We also highlighted the mechanisms, targets, technical issues, and patterns of CL-taVNS. By reviewing, proposing, and highlighting, this work might draw a preliminary blueprint for the development of CL-taVNS.

## Introduction

### Timing: From VNS to CL-taVNS

Vagal nerve stimulation (VNS) was initially a non-invasive neuromodulation technique with a history that can be traced back to the 1880s (Lanska, 2002). However, throughout the 20th century, studies on stimulation of the vagal nerve were almost inseparable from the characteristic of invasiveness (Thompson et al., 2021). Intriguingly, by the end of the second millennium, transcutaneous auricular vagal nerve stimulation (taVNS), an authentic non-invasive brain stimulation (NIBS), had emerged (Ventureyra, 2000). Inspired by neuroanatomy [the distribution of the auricular branch of the vagal nerve (ABVN) in the auricular concha], auricular acupuncture (AA), and invasive VNS (iVNS), the concept of taVNS opened a new era in the field of neuromodulation (Wang et al., 2021), particularly having a similar pattern of activation with the iVNS (Badran et al., 2018). In 2020, further progress was made based on taVNS, with researchers officially proposing the closed-loop taVNS (CL-taVNS; Badran et al., 2020; Cook et al., 2020).

### Anatomical Basis, Mechanisms, and Indications of taVNS

The ABVN is directly connected to the nucleus tractus solitarii (NTS) in the medulla, which is the endpoint of the afferent fibers of the vagal nerve and is recognized as a relay station for visceral sensation, plays a relay role in receiving signals from the ear, and adjusts the function of the body (Schachter and Saper, 1998). The NTS makes forward projections directly or indirectly to locus ceruleus, hypothalamus, thalamus, amygdala, hippocampus, and prefrontal cortex (Ricardo and Koh, 1978; Ter Horst et al., 1989; Van Eden and Buijs, 2000; Castle et al., 2005), with the release of neurotransmitters including norepinephrine (NE), serotonin (5-HT) and dopamine (DA; Badran et al., 2018). Efferent fibers in the vagal nerve can control multiple peripheral organs (Wang et al., 2021), including the heart, lungs, liver, stomach, pancreas, and kidneys (Moini and Piran, 2020). Therefore, taVNS has confirmed and potential applications for various diseases related to the central and peripheral nervous systems.

Principally by balancing the autonomic nervous system (increasing parasympathetic activity and reducing sympathetic activity; Deuchars et al., 2018), taVNS has several indications in the brain, cardiovascular, and digestive system diseases (Wang et al., 2021). In addition, taVNS has potential benefits for type 2 diabetes (T2D; Wang et al., 2015), obesity (Yu et al., 2021), and rheumatoid arthritis (RA; Addorisio et al., 2019). Due to the anatomical properties of the vagal nerve and the major mechanism of taVNS, more indications of taVNS may emerge in the future.

### From Open-Loop to Closed-Loop taVNS

Some of these taVNS indications are associated with clinically detectable, altered dynamics, and the aberrant activity, in principle, can be restored through taVNS. Moreover, these abnormal patterns occur intermittently and sometimes unpredictably. Thus, it is necessary to modify the original taVNS from open-loop to closed-loop to correct the anomaly at an early stage.

The CL-taVNS systems adapt rapidly to changing conditions and thus offer a personalized taVNS for individualized control with increased therapeutic efficiency, improved quality of life, and reduced severity of side effects (Kaniusas et al., 2019a). A brief definition of CL-taVNS might be an automatic control taVNS system in which its process is regulated by biofeedback signal(s). As a result, a CL-taVNS system should primarily include a biosignal(s) sensor (identifier) and a taVNS stimulator integrated with remote-control solutions (Kaniusas et al., 2019a). For instance, in the existing CL-taVNS systems, behavioral changes are the biomarkers for switching the stimulation process (Napadow et al., 2012; Garcia et al., 2017; Badran et al., 2020; Cook et al., 2020). It can be inferred that other biomarkers may also be available for developing new CL-taVNS systems and multiple types of CL-taVNS systems triggered by specific biomarkers and leaving other functions unaffected are therefore desirable.

### Disease-Oriented Development of CL-taVNS

**Figure 1:**
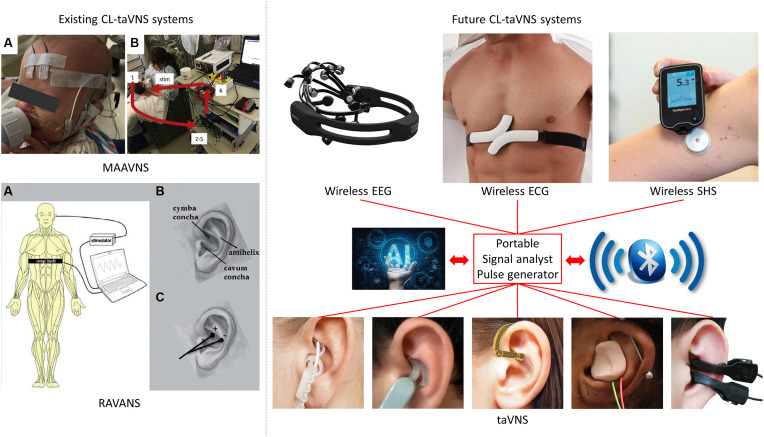
Illustration of two known and three future CL-taVNS systems. MAAVNS (image from https://doi.org/10.1016/j.brs.2020.02.028): motor-activated auricular vagus nerve stimulation. **(A)** EMG lead placement: active lead on buccinator, reference lead on the frontal eminence, ground lead in the center of the forehead. **(B)** Overview of MAAVNS setup: EMG signals from facial muscles (1) were processed (2–5) and used to trigger stimulation (6, stim). RAVANS (image from https://doi.org/10.1111/j.1526-4637.2012.01385.x): respiratory-gated auricular vagal afferent nerve stimulation. **(A)** Subjects were outfitted with a thoracic belt to measure respiratory excursions. This signal was transduced and fed into a laptop controller, allowing for left transcutaneous auricular vagal nerve stimulation to occur only during the expiratory phase of respiration. **(B)** Auricular anatomy includes essential regions, including the cymba and cavum conchae, as well as the antihelix. **(C)** Auricular electrodes were placed within the cymba concha and antihelix, the two regions found to be most consistently innervated by the auricular branch of the vagal nerve. EEG-gated taVNS, EEG: electroencephalography, including the use of artificial intelligence (AI) and Bluetooth. ECG-gated taVNS, ECG: electrocardiography, including the use of artificial intelligence (AI) and Bluetooth. SHS-gated taVNS, SHS: subcutaneous humoral signals, including the use of artificial intelligence (AI) and Bluetooth. taVNS: transcutaneous auricular vagal nerve stimulation.

The development of CL-taVNS systems may be diverse, but we consider that they should be disease-oriented. Thus, in addition to a short review of the known CL-taVNS systems, three future putative applications of the technique are suggested in this article (Figure 1) which aims to inform the development of CL-taVNS from a clinical perspective.

## Existing CL-taVNS Systems

### Motor-Activated Auricular Vagus Nerve Stimulation (MAAVNS)

The field of CL-taVNS was pioneered by Badran et al. (2019), with an abstract in which the authors described their method as electromyography (EMG)-gated CL-taVNS. They followed up the study and formally proposed the CL-taVNS in 2020 (Cook et al., 2020) and named it motor-activated auricular vagus nerve stimulation (MAAVNS). MAAVNS pairs taVNS with motor activity, delivering taVNS during targeted motor activity (Cook et al., 2020). Their studies demonstrated that MAAVNS is a promising neurorehabilitation tool in neonates (Badran et al., 2020; Cook et al., 2020). MAAVNS has been translated to adult upper limb rehabilitation, and this application is being investigated in a small randomized trial (ClinicalTrials.gov Identifier: NCT04129242).

### Respiratory-Gated Auricular Vagal Afferent Nerve Stimulation (RAVANS)

Another form of CL-taVNS proposed much earlier than MAAVNS is respiratory-gated auricular vagal afferent nerve stimulation (RAVANS). RAVANS works on the principle that inhalation induces transient inhibition of vagal nerve activity (Thompson et al., 2021). Positive results were recorded when RAVANS was applied in pain subjects, including pelvic pain (Napadow et al., 2012) and migraine (Garcia et al., 2017). Furthermore, although no firm conclusions have been drawn, it seemed that RAVANS might also lower blood pressure in hypertensive patients (Fisher et al., 2018; Stowell et al., 2019). Significantly, in healthy subjects, expiratory-gated and non-respiratory-gated taVNS exert apparent cardioinhibitory effects with high pre-stimulatory heart rate, whereas inspiratory-gated taVNS does not affect heart rate (Paleczny et al., 2021).

## Future CL-taVNS Systems

MAAVNS uses EMG to trigger the taVNS procedure, while RAVANS applies mechanical signals with subjects outfitted with a thoracic belt to measure respiratory excursions (Figure 1). However, electroencephalographic (EEG) and electrocardiographic (ECG) signals should not be ignored. Moreover, since taVNS is a potential treatment option for T2D and RA, subcutaneous humoral signals (SHS) may be an option to trigger taVNS in specific patients. Therefore, in the following sections, we propose three potential CL-taVNS systems.

### EEG-Gated CL-taVNS

Clinical evidence indicates that taVNS is an effective treatment for epilepsy (Rong et al., 2014) and major depressive disorder (MDD; Fang et al., 2016; Rong et al., 2016). EEG is the most specific method to define the epileptogenic cortex (Noachtar and Rémi, 2009). Meanwhile, the EEG-based computer-aided technique may be a suitable clinical diagnostic tool for MDD (Mumtaz et al., 2017). Therefore, we speculate that it is possible to develop an EEG-gated CL-taVNS system. In such a system, detection of an abnormal EEG signal would immediately activate ABVN stimulation to alleviate symptoms. For example, spike-and-wave patterns, typically arising from complex interactions between thalamic and neocortical neurons, are the hallmark of generalized absence seizure (Berényi et al., 2012), and may be an apposite EEG biomarker for epilepsy and could provide a trigger for EEG-gated CL-taVNS as a treatment for epilepsy. A recent replication study supports the diagnostic value of EEG-vigilance regulation and its usefulness as a biomarker for treatment choice in MDD (Ip et al., 2021), which can also be a candidate for switching EEG-gated CL-taVNS in treating MDD.

In addition to epilepsy and MDD, a myriad of neurological and psychiatric disorders have event-related EEG biomarkers. Therefore, EEG-gated CL-taVNS may be extended to more brain diseases in the future, including but not limited to Alzheimer’s disease (AD; Chang et al., 2018; Gaubert et al., 2019), and ischaemic stroke (Ajčević et al., 2021; Dawson et al., 2021). A more in-depth systematic review should focus primarily on the EEG biomarkers of the taVNS-ameliorable brain diseases to explore potential therapeutic applications.

Previously, ear-EEG-gated CL-taVNS has been proposed to modulate attention (Ruhnau and Zaehle, 2021); however, its apparent limitations and challenges make it seem not practical. Hence, we suggest that EEG-gated CL-taVNS optimized from the original EEG headset is a more promising form (Figure 1).

### ECG-Gated CL-taVNS

Cardiovascular diseases are often treated on the basis of inhibiting the over-excitation of the sympathetic system by pharmacological interventions, while vagal modulation has been largely ignored (Liu et al., 2019). The vagal nerve provides the primary parasympathetic innervation to the heart. Due to the critical VNS mechanism (reducing sympathetic activity and increasing parasympathetic activity), VNS may be a therapeutic approach for chronic heart failure (De Ferrari et al., 2010). Preclinical studies applying taVNS [low-level tragus stimulation (LLTS)] in canines and rodents have shown promising results in suppressing atrial fibrillation, alleviating post-myocardial infarction, ventricular arrhythmias, and ischemia-reperfusion injury along with improving diastolic parameters in heart failure with preserved left ventricular ejection fraction (Jiang et al., 2020). Preliminary pilot clinical studies using taVNS with low-level tragus stimulation in patients with the above heart conditions have demonstrated promising results (Jiang et al., 2020). These include suppression of atrial fibrillation (Stavrakis et al., 2015, 2020), reduction of myocardial ischemia-reperfusion injury in patients with ST-segment elevation myocardial infarction (STEMI; Yu et al., 2017), antianginal effect (Zamotrinsky et al., 1997), amelioration of left ventricular strain (Tran et al., 2019), and improved endothelial function in patients with heart failure with reduced ejection fraction (Dasari et al., 2018).

ECG is a reliable tool for monitoring heart diseases. Thus, the development of an ECG-gated CL-taVNS is important and imminent. Such a system might work as follows: when an irregular ECG signal is detected, immediate taVNS may help to reduce or terminate the anomaly. The current wearable ECG device might be heavy and uncomfortable (Figure 1), but it is clear that a more lightweight device can be designed.

We speculate that ECG-gated CL-taVNS might be available for only a portion of patients with cardiovascular problems. Therefore, like EEG-gated CL-taVNS, a more in-depth systematic review of ECG biomarkers in cardiovascular problems amenable or otherwise to taVNS is urgently needed to inform the development of the ECG-gated CL-taVNS.

### SHS-Gated CL-taVNS

SHS-gated CL-taVNS can be developed for the treatment of T2D and RA, with the capacity to detect abnormally elevated blood glucose or cytokine levels (Figure 1).

VNS or taVNS has potential applications in treating T2D (Johnson and Wilson, 2018). However, preclinical results are controversial. VNS has been shown to increase blood glucose levels in non-diabetic rats (Meyers et al., 2016; Stauss et al., 2018), but VNS or taVNS reduced levels in diabetic rats (Wang et al., 2015; Yin et al., 2019). These results suggest that the beneficial effects of VNS or taVNS on blood glucose levels are only apparent in the context of diabetes. Thus, it is essential to develop a blood-glucose-gated CL-taVNS which on detecting hyperglycemia would activate taVNS to reduce and stabilize the levels.

Clinical evidence shows that taVNS attenuates systemic inflammatory responses in RA patients (Addorisio et al., 2019). Thus, it is also possible to develop a cytokine-gated CL-taVNS system which would inhibit inflammatory responses on detection of abnormal cytokine levels. This system might also be applicable to other autoimmune disorders, such as systemic lupus erythematosus (Aranow et al., 2021) and Sjögren syndrome.

To our knowledge, SHS-gated CL-taVNS may also extend to other diseases, so other SHS-altered-associated internal conditions, which are reversible by taVNS, also merit a systematic review.

## Discussion

### The Electroceuticals Through the Vagal Nerve

The vagal nerve offers an alternative means to modify brain and other organ functions *via* artificial stimulation (Moore, 2015). In recent years, electrical stimulation of the vagal nerve has progressively come into focus as a non-pharmaceutical or electroceutical treatment option for various diseases (Kaniusas et al., 2019b). Therefore, both invasive and non-invasive VNS have gained particular interest worldwide (Kaniusas et al., 2019a).

### The Potential of CL-taVNS

Since its clinical applications began in the 1990s, iVNS has become a pioneering tool of vagal modulation (Moore, 2015), and 20 years of development of taVNS has refined this remarkable tool (Ventureyra, 2000). Given that other closed-loop neuromodulation studies, such as closed-loop transcranial electrical stimulation (CL-TES; Berényi et al., 2012) and closed-loop transcranial alternating current stimulation (CL-tACS; Brittain et al., 2013), show exciting results, the potential of CL-taVNS is clear, even in its infancy.

### Artifacts of the Proposed CL-taVNS Systems

It is worth noting that due to the electrical peculiarities of tACS, the artifacts of tACS make it difficult (although technically possible) to parse EEG signals during stimulation (Wu et al., 2021) but viable only in intermittent closed-loop stimulation protocol, in which EEG recordings are performed before and after stimulation (Stecher et al., 2021). However, EEG-gated CL-taVNS is unaffected by these artifacts, so it will not have such a technical limitation. Also, it can be inferred that ECG-gated CL-taVNS and SHS-gated CL-taVNS will not have the same problem due to their technical properties.

### Artificial Intelligence (AI) and Bluetooth in CL-taVNS and the Wearable Devices

The known CL-taVNS systems, RAVANS (Napadow et al., 2012; Garcia et al., 2017) and MAAVNS (Badran et al., 2020; Cook et al., 2020), majorly pair with behaviors, while the future CL-taVNS systems we propose rely predominantly on objective indices, such as EEG, ECG, blood glucose levels, and cytokine levels. Therefore, artificial intelligence (AI) with specific algorithms might be needed for these CL-taVNS systems. Furthermore, wireless EEG, ECG, SHS, and taVNS devices are becoming popular, and Bluetooth or similar technologies should be used for communication among these wearable devices (Figure 1).

The future CL-taVNS systems we have proposed should primarily target some ongoing EEG/ECG/SHS activity biomarkers, such as the spike-and-wave patterns of epilepsy (EEG; Berényi et al., 2012) and the ST-segment elevation of myocardial infarction (ECG; Jiang et al., 2020). These biomarkers, which are condition-specific, should be identified automatically by AI, which would then immediately trigger the taVNS process to relieve the symptoms.

### On-Off Biomarkers of CL-taVNS

The easiest way to close the loop is to provide a simple on-demand activation of taVNS *via* subjective biofeedback or via a biomarker from the patient (Kaniusas et al., 2019a). In all the above CL-taVNS systems, motor changes (RAVANS and MAAVNS) and abnormal EEG/ECG/SHS features are or will be employed as the biomarkers (On Biomarkers) which trigger the CL-taVNS systems. However, there is still a lack of confirmed biomarkers (Off Biomarkers) to turn off the CL-taVNS systems. End or reversal of the motor changes and normalized EEG/ECG/SHS features themselves might be possible Off Biomarkers. In addition to these, five potential neurophysiological biomarkers of taVNS include heart rate variability (which can be extracted from ECG data), vagal sensory evoked potentials, pupil diameter, event related potentials (ERP, especially P300), and salivary alpha-amylase secretion, which have been proposed in a narrative review (Burger et al., 2020). While the efficacy of taVNS biomarkers is controversial, pupil size in scotopic illumination with taVNS at 2 mA may be a reliable and non-invasive biomarker of vagal activation and could be used as a user-friendly online indicator of the stimulation’s effectiveness (Capone et al., 2021) and as an Off Biomarker of CL-taVNS.

### Instantaneous CL-taVNS

**Figure 2:**
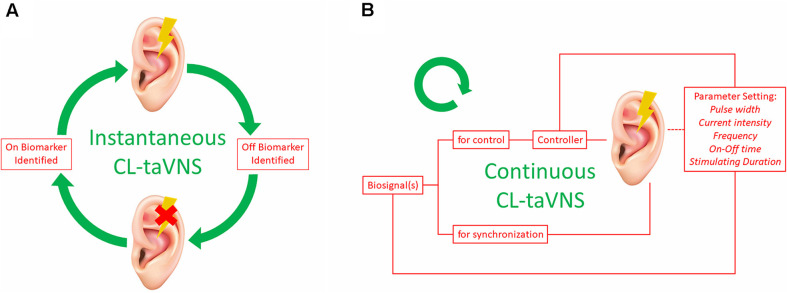
Two Patterns of CL-taVNS. **(A)** workflow of instantaneous CL-taVNS. The system will be activated as soon as the On Biomarker is identified and deactivated when the Off Biomarker is determined by the identifier simultaneously. **(B)** Workflow of continuous CL-taVNS. The biofeedback is used to control stimulation parameters of taVNS (pulse width, current intensity, frequency, On-Off time, and stimulating duration) to adhere to momentary therapeutic needs. The biofeedback can also be used for temporal synchronization of the applied stimuli with inner body rhythms to interfere constructively with the dynamics of the body. CL-taVNS: Closed-loop transcutaneous auricular vagal nerve stimulation.

For some instant conditions, such as generalized absence seizure, instantaneous stimulation of the ABVN may suffice to eliminate the problem. The required stimulation may be short-term, and with the use of On-Off biomarkers, CL-taVNS may form a loop (Figure 2A). Briefly speaking, the system is activated and deactivated immediately with On and Off biomarkers, respectively. The existing CL-taVNS systems (RAVANS and MAAVNS) have already achieved the pattern successfully. However, additional studies are required to validate the feasibility of this workflow in the future CL-taVNS systems we proposed.

### Continuous CL-taVNS

Continuous CL-taVNS has advantages for some sustained conditions such as hyperglycemia. Recording and analysis of biosignal(s) in response to taVNS may close the loop and thus allow optimization and personalization of taVNS therapy (Kaniusas et al., 2019a). The biosignal(s) should signal the controller to adjust the stimulus pattern and synchronize the stimulus. According to the biosignal changes, the controller cyclically modifies the parameter settings of taVNS (Figure 2B), including pulse width, current intensity, frequency, On-Off time, and stimulating duration (Thompson et al., 2021). Since the individual human body as the system to be controlled is never sufficiently known and is subjected to continuous changes over time, adaptive methods (such as AI and machine learning) might be used to define the controller (Kaniusas et al., 2019a).

## Summary

Optimizing from taVNS, CL-taVNS, which was formally proposed in 2020, has become a novel direction of neuromodulation. This work reviewed two known forms of CL-taVNS and proposed three future approaches. Significantly, the mechanisms, targets, technical issues, patterns of CL-taVNS, and motivations to move the method from open-loop to closed-loop were introduced and discussed. There is much room for improvement as CL-taVNS continues to emerge as a promising neuromodulation modality.

## Data Availability Statement

The original contributions presented in the study are included in the article, further inquiries can be directed to the corresponding author/s.

## Author Contributions

All authors contributed to the content of this work, edited the manuscript, and approved the submission.

## Conflict of Interest

The authors declare that the research was conducted in the absence of any commercial or financial relationships that could be construed as a potential conflict of interest.

## Publisher’s Note

All claims expressed in this article are solely those of the authors and do not necessarily represent those of their affiliated organizations, or those of the publisher, the editors and the reviewers. Any product that may be evaluated in this article, or claim that may be made by its manufacturer, is not guaranteed or endorsed by the publisher.

## References

[B1] Addorisio M. E. Imperato G. H. de Vos A. F. Forti S. Goldstein R. S. Pavlov V. A.. (2019). Investigational treatment of rheumatoid arthritis with a vibrotactile device applied to the external ear. Bioelectron. Med. 5:4. 10.1186/s42234-019-0020-4 32232095 PMC7098240

[B2] Ajčević M. Furlanis G. Miladinović A. Buoite Stella A. Caruso P. Ukmar M.. (2021). Early EEG alterations correlate with CTP hypoperfused volumes and neurological deficit: a wireless EEG study in hyper-acute ischemic stroke. Ann. Biomed. Eng. 49, 2150–2158. 10.1007/s10439-021-02735-w 33604799 PMC8455382

[B3] Aranow C. Atish-Fregoso Y. Lesser M. Mackay M. Anderson E. Chavan S.. (2021). Transcutaneous auricular vagus nerve stimulation reduces pain and fatigue in patients with systemic lupus erythematosus: a randomised, double-blind, sham-controlled pilot trial. Ann. Rheum. Dis. 80, 203–208. 10.1136/annrheumdis-2020-217872 33144299

[B5] Badran B. W. Dowdle L. T. Mithoefer O. J. LaBate N. T. Coatsworth J. Brown J. C.. (2018). Neurophysiologic effects of transcutaneous auricular vagus nerve stimulation (taVNS) *via* electrical stimulation of the tragus: a concurrent taVNS/fMRI study and review. Brain Stimul. 11, 492–500. 10.1016/j.brs.2017.12.009 29361441 PMC6487660

[B6] Badran B. W. Jenkins D. D. Cook D. Thompson S. Dancy M. DeVries W. H.. (2020). Transcutaneous auricular vagus nerve stimulation-paired rehabilitation for oromotor feeding problems in newborns: an open-label pilot study. Front. Hum. Neurosci. 14:77. 10.3389/fnhum.2020.00077 32256328 PMC7093597

[B4] Badran B. Jenkins D. DeVries W. Dancy M. Cook D. Mappin G.. (2019). Development of closed-loop transcutaneous auricular vagus nerve stimulation (taVNS) as a neurorehabilitation tool. Brain Stimul. 12:523. 10.1016/j.brs.2018.12.721

[B7] Berényi A. Belluscio M. Mao D. Buzsáki G. (2012). Closed-loop control of epilepsy by transcranial electrical stimulation. Science 337, 735–737. 10.1126/science.1223154 22879515 PMC4908579

[B8] Brittain J.-S. Probert-Smith P. Aziz T. Z. Brown P. (2013). Tremor suppression by rhythmic transcranial current stimulation. Curr. Biol. 23, 436–440. 10.1016/j.cub.2013.01.068 23416101 PMC3629558

[B9] Burger A. M. D’Agostini M. Verkuil B. Van Diest I. (2020). Moving beyond belief: a narrative review of potential biomarkers for transcutaneous vagus nerve stimulation. Psychophysiology 57:e13571. 10.1111/psyp.13571 32202671

[B10] Capone F. Motolese F. Di Zazzo A. Antonini M. Magliozzi A. Rossi M.. (2021). The effects of transcutaneous auricular vagal nerve stimulation on pupil size. Clin. Neurophysiol. 132, 1859–1865. 10.1016/j.clinph.2021.05.014 34147923

[B11] Castle M. Comoli E. Loewy A. (2005). Autonomic brainstem nuclei are linked to the hippocampus. Neuroscience 134, 657–669. 10.1016/j.neuroscience.2005.04.031 15975727

[B12] Chang C.-H. Lane H.-Y. Lin C.-H. (2018). Brain stimulation in Alzheimer’s disease. Front. Psychiatry 9:201. 10.3389/fpsyt.2018.00201 29910746 PMC5992378

[B13] Cook D. N. Thompson S. Stomberg-Firestein S. Bikson M. George M. S. Jenkins D. D.. (2020). Design and validation of a closed-loop, motor-activated auricular vagus nerve stimulation (MAAVNS) system for neurorehabilitation. Brain Stimul. 13, 800–803. 10.1016/j.brs.2020.02.028 32289710 PMC7196027

[B14] Dasari T. W. Gabor F. Csipo T. Palacios F. S. Yabluchanskiy A. Samannan R.. (2018). Non-invasive neuromodulation of vagus activity improves endothelial function in patients with heart failure with reduced ejection fraction: a randomized study. J. Cardiac Failure 24, S59–S60. 10.1016/j.cardfail.2018.07.266

[B15] Dawson J. Liu C. Y. Francisco G. E. Cramer S. C. Wolf S. L. Dixit A.. (2021). Vagus nerve stimulation paired with rehabilitation for upper limb motor function after ischaemic stroke (VNS-REHAB): a randomised, blinded, pivotal, device trial. Lancet 397, 1545–1553. 10.1016/S0140-6736(21)00475-X 33894832 PMC8862193

[B16] De Ferrari G. M. Crijns H. J. G. M. Borggrefe M. Milasinovic G. Smid J. Zabel M.. (2010). Chronic vagus nerve stimulation: a new and promising therapeutic approach for chronic heart failure. Eur. Heart J. 32, 847–855. 10.1093/eurheartj/ehq391 21030409

[B17] Deuchars S. A. Lall V. K. Clancy J. Mahadi M. Murray A. Peers L.. (2018). Mechanisms underpinning sympathetic nervous activity and its modulation using transcutaneous vagus nerve stimulation. Exp. Physiol. 103, 326–331. 10.1113/EP086433 29205954 PMC5887928

[B18] Fang J. Rong P. Hong Y. Fan Y. Liu J. Wang H.. (2016). Transcutaneous vagus nerve stimulation modulates default mode network in major depressive disorder. Biol. Psychiatry 79, 266–273. 10.1016/j.biopsych.2015.03.025 25963932 PMC4838995

[B19] Fisher H. Stowell J. Garcia R. Sclocco R. Goldstein J. Napadow V.. (2018). “Acute effects of respiratory-gated auricular vagal afferent nerve stimulation (RAVANS) in the modulation of blood pressure in hypertensive patients,” in 2018 Computing in Cardiology Conference (CinC), Vol. 45, 1–4. 10.22489/CinC.2018.346

[B20] Garcia R. G. Lin R. L. Lee J. Kim J. Barbieri R. Sclocco R.. (2017). Modulation of brainstem activity and connectivity by respiratory-gated auricular vagal afferent nerve stimulation (RAVANS) in migraine patients. Pain 158, 1461–1472. 10.1097/j.pain.0000000000000930 28541256 PMC5517046

[B21] Gaubert S. Raimondo F. Houot M. Corsi M.-C. Naccache L. Diego Sitt J.. (2019). EEG evidence of compensatory mechanisms in preclinical Alzheimer’s disease. Brain 142, 2096–2112. 10.1093/brain/awz150 31211359

[B22] Ip C.-T. Ganz M. Dam V. H. Ozenne B. Rüesch A. Köhler-Forsberg K.. (2021). NeuroPharm study: EEG wakefulness regulation as a biomarker in MDD. J. Psychiatr. Res. 141, 57–65. 10.1016/j.jpsychires.2021.06.021 34175743

[B23] Jiang Y. Po S. S. Amil F. Dasari T. W. (2020). Non-invasive low-level tragus stimulation in cardiovascular diseases. Arrhythm. Electrophysiol. Rev. 9, 40–46. 10.15420/aer.2020.01 32637119 PMC7330730

[B24] Johnson R. L. Wilson C. G. (2018). A review of vagus nerve stimulation as a therapeutic intervention. J. Inflamm. Res. 11, 203–213. 10.2147/JIR.S163248 29844694 PMC5961632

[B25] Kaniusas E. Kampusch S. Tittgemeyer M. Panetsos F. Gines R. F. Papa M.. (2019a). Current directions in the auricular vagus nerve stimulation II - an engineering perspective. Front. Neurosci. 13:772. 10.3389/fnins.2019.00772 31396044 PMC6667675

[B26] Kaniusas E. Kampusch S. Tittgemeyer M. Panetsos F. Gines R. F. Papa M.. (2019b). Current directions in the auricular vagus nerve stimulation I - a physiological perspective. Front. Neurosci. 13:854. 10.3389/fnins.2019.00854 31447643 PMC6697069

[B27] Lanska D. J. (2002). JL Corning and vagal nerve stimulation for seizures in the 1880s. Neurology 58, 452–459. 10.1212/wnl.58.3.452 11839848

[B28] Liu L. Zhao M. Yu X. Zang W. (2019). Pharmacological modulation of vagal nerve activity in cardiovascular diseases. Neurosci. Bull. 35, 156–166. 10.1007/s12264-018-0286-7 30218283 PMC6357265

[B29] Meyers E. E. Kronemberger A. Lira V. Rahmouni K. Stauss H. M. (2016). Contrasting effects of afferent and efferent vagal nerve stimulation on insulin secretion and blood glucose regulation. Physiol. Rep. 4:e12718. 10.14814/phy2.12718 26884478 PMC4759047

[B30] Moini J. Piran P. (2020). “Chapter 10 - Cranial nerves,” in Functional and Clinical Neuroanatomy, eds Moini J. Piran P. (Academic Press), 319–344. 10.1016/B978-0-12-817424-1.00010-0

[B31] Moore S. (2015). The Vagus Nerve: A Back Door for Brain Hacking. New York, NY: IEEE Spectrum.

[B32] Mumtaz W. Xia L. Ali S. S. A. Yasin M. A. M. Hussain M. Malik A. S. (2017). Electroencephalogram (EEG)-based computer-aided technique to diagnose major depressive disorder (MDD). Biomed. Signal Process. Control 31, 108–115. 10.1016/j.bspc.2016.07.006

[B33] Napadow V. Edwards R. R. Cahalan C. M. Mensing G. Greenbaum S. Valovska A.. (2012). Evoked pain analgesia in chronic pelvic pain patients using respiratory-gated auricular vagal afferent nerve stimulation. Pain Med. 13, 777–789. 10.1111/j.1526-4637.2012.01385.x 22568773 PMC3376238

[B34] Noachtar S. Rémi J. (2009). The role of EEG in epilepsy: a critical review. Epilepsy Behav. 15, 22–33. 10.1016/j.yebeh.2009.02.035 19248841

[B35] Paleczny B. Seredyński R. Ponikowska B. (2021). Inspiratory-and expiratory-gated transcutaneous vagus nerve stimulation have different effects on heart rate in healthy subjects: preliminary results. Clin. Auton. Res. 31, 205–214. 10.1007/s10286-019-00604-0 30941526 PMC8041682

[B36] Ricardo J. A. Koh E. T. (1978). Anatomical evidence of direct projections from the nucleus of the solitary tract to the hypothalamus, amygdala and other forebrain structures in the rat. Brain Res. 153, 1–26. 10.1016/0006-8993(78)91125-3 679038

[B37] Rong P. Liu J. Wang L. Liu R. Fang J. Zhao J.. (2016). Effect of transcutaneous auricular vagus nerve stimulation on major depressive disorder: a nonrandomized controlled pilot study. J. Affect. Dis. 195, 172–179. 10.1016/j.jad.2016.02.031 26896810 PMC4828906

[B38] Rong P. Liu A. Zhang J. Wang Y. He W. Yang A.. (2014). Transcutaneous vagus nerve stimulation for refractory epilepsy: a randomized controlled trial. Clin. Sci. (Lond) 10.1042/CS20130518. [Online ahead of print]. 24684603

[B39] Ruhnau P. Zaehle T. (2021). Transcranial auricular vagus nerve stimulation (taVNS) and ear-EEG: potential for closed-loop portable non-invasive brain stimulation. Front. Hum. Neurosci. 15:699473. 10.3389/fnhum.2021.699473 34194308 PMC8236702

[B40] Schachter S. C. Saper C. B. (1998). Vagus nerve stimulation. Epilepsia 39, 677–686. 10.1111/j.1528-1157.1998.tb01151.x 9670894

[B41] Stauss H. M. Stangl H. Clark K. C. Kwitek A. E. Lira V. A. (2018). Cervical vagal nerve stimulation impairs glucose tolerance and suppresses insulin release in conscious rats. Physiol. Rep. 6:e13953. 10.14814/phy2.13953 30569658 PMC6300710

[B42] Stavrakis S. Humphrey M. B. Scherlag B. J. Hu Y. Jackman W. M. Nakagawa H.. (2015). Low-level transcutaneous electrical vagus nerve stimulation suppresses atrial fibrillation. J. Am. Coll. Cardiol. 65, 867–875. 10.1016/j.jacc.2014.12.026 25744003 PMC4352201

[B43] Stavrakis S. Stoner J. A. Humphrey M. B. Morris L. Filiberti A. Reynolds J. C.. (2020). TREAT AF (transcutaneous electrical vagus nerve stimulation to suppress atrial fibrillation): a randomized clinical trial. JACC Clin. Electrophysiol. 6, 282–291. 10.1016/j.jacep.2019.11.008 32192678 PMC7100921

[B44] Stecher H. I. Notbohm A. Kasten F. H. Herrmann C. S. (2021). A comparison of closed loop vs. fixed frequency tACS on modulating brain oscillations and visual detection. Front. Hum. Neurosci. 15:661432. 10.3389/fnhum.2021.661432 34248524 PMC8261289

[B45] Stowell J. Garcia R. G. Staley R. Sclocco R. Fisher H. Napadow V.. (2019). “Dose-optimization of respiratory-gated auricular vagal afferent nerve stimulation (RAVANS) for blood pressure modulation in hypertensive patients,” in 2019 Computing in Cardiology Conference (CinC), (IEEE), Vol. 46, 1–4. 10.22489/CinC.2019.098

[B46] Ter Horst G. De Boer P. Luiten P. Van Willigen J. (1989). Ascending projections from the solitary tract nucleus to the hypothalamus. A phaseolus vulgaris lectin tracing study in the rat. Neuroscience 31, 785–797. 10.1016/0306-4522(89)90441-7 2594200

[B47] Thompson S. L. O’Leary G. H. Austelle C. W. Gruber E. Kahn A. T. Manett A. J.. (2021). A Review of parameter settings for invasive and non-invasive Vagus Nerve Stimulation (VNS) applied in neurological and psychiatric disorders. Front. Neurosci. 15:709436. 10.3389/fnins.2021.709436 34326720 PMC8313807

[B48] Tran N. Asad Z. Elkholey K. Scherlag B. J. Po S. S. Stavrakis S. (2019). Autonomic neuromodulation acutely ameliorates left ventricular strain in humans. J. Cardiovasc. Transl. Res. 12, 221–230. 10.1007/s12265-018-9853-6 30560316 PMC6579714

[B49] Van Eden C. G. Buijs R. M. (2000). “Functional neuroanatomy of the prefrontal cortex: autonomic interactions,” in Progress in Brain Research, (Elsevier), 49–62. 10.1016/S0079-6123(00)26006-8 11105639

[B50] Ventureyra E. C. (2000). Transcutaneous vagus nerve stimulation for partial onset seizure therapy. Childs Nerv. Syst. 16, 101–102. 10.1007/s003810050021 10663816

[B52] Wang Y. Li S.-Y. Wang D. Wu M.-Z. He J.-K. Zhang J.-L.. (2021). Transcutaneous auricular vagus nerve stimulation: from concept to application. Neurosci. Bull. 37, 853–862. 10.1007/s12264-020-00619-y 33355897 PMC8192665

[B51] Wang S. Zhai X. Li S. McCabe M. F. Wang X. Rong P. (2015). Transcutaneous vagus nerve stimulation induces tidal melatonin secretion and has an antidiabetic effect in zucker fatty rats. PLoS One 10:e0124195. 10.1371/journal.pone.0124195 25880500 PMC4400163

[B53] Wu L. Liu T. Wang J. (2021). Improving the effect of transcranial alternating current stimulation (tACS): a systematic review. Front. Hum. Neurosci. 15:652393. 10.3389/fnhum.2021.652393 34163340 PMC8215166

[B54] Yin J. Ji F. Gharibani P. Chen J. D. (2019). Vagal nerve stimulation for glycemic control in a rodent model of type 2 diabetes. Obes. Surg. 29, 2869–2877. 10.1007/s11695-019-03901-9 31222497 PMC10461220

[B56] Yu Y. He X. Zhang J. Tang C. Rong P. (2021). Transcutaneous auricular vagal nerve stimulation inhibits hypothalamic P2Y1R expression and attenuates weight gain without decreasing food intake in Zucker diabetic fatty rats. Sci. Prog. 104:00368504211009669. 10.1177/00368504211009669 33848220 PMC10358456

[B55] Yu L. Huang B. Po S. S. Tan T. Wang M. Zhou L.. (2017). Low-level tragus stimulation for the treatment of ischemia and reperfusion injury in patients with ST-segment elevation myocardial infarction: a proof-of-concept study. JACC Cardiovasc. Interv. 10, 1511–1520. 10.1016/j.jcin.2017.04.036 28797427

[B57] Zamotrinsky A. Afanasiev S. Karpov R. S. Cherniavsky A. (1997). Effects of electrostimulation of the vagus afferent endings in patients with coronary artery disease. Coron. Artery Dis. 8, 551–557. 9431484

